# A Genome-Wide Association Study Identifies Novel and Functionally Related Susceptibility Loci for Kawasaki Disease

**DOI:** 10.1371/journal.pgen.1000319

**Published:** 2009-01-09

**Authors:** David Burgner, Sonia Davila, Willemijn B. Breunis, Sarah B. Ng, Yi Li, Carine Bonnard, Ling Ling, Victoria J. Wright, Anbupalam Thalamuthu, Miranda Odam, Chisato Shimizu, Jane C. Burns, Michael Levin, Taco W. Kuijpers, Martin L. Hibberd, International Kawasaki Disease Genetics Consortium

**Affiliations:** 1 School of Pediatrics and Child Health, University of Western Australia, Perth, Australia; 2 Infectious Diseases, Genome Institute of Singapore, Singapore, Singapore; 3 Division of Pediatric Hematology, Immunology, and Infectious Diseases, Emma Children's Hospital Academic Medical Center, Amsterdam, The Netherlands; 4 Human Genetics Programme, Genome Institute of Singapore, Singapore, Singapore; 5 Department of Pediatrics, Division of Medicine, Imperial College London, London, United Kingdom; 6 Department of Pediatrics, University of California San Diego School of Medicine, Rady Children's Hospital, San Diego, California, United States of America; The University of Queensland, Australia

## Abstract

Kawasaki disease (KD) is a pediatric vasculitis that damages the coronary arteries in 25% of untreated and approximately 5% of treated children. Epidemiologic data suggest that KD is triggered by unidentified infection(s) in genetically susceptible children. To investigate genetic determinants of KD susceptibility, we performed a genome-wide association study (GWAS) in 119 Caucasian KD cases and 135 matched controls with stringent correction for possible admixture, followed by replication in an independent cohort and subsequent fine-mapping, for a total of 893 KD cases plus population and family controls. Significant associations of 40 SNPs and six haplotypes, identifying 31 genes, were replicated in an independent cohort of 583 predominantly Caucasian KD families, with *NAALADL2* (rs17531088, *p*_combined_ = 1.13×10^−6^) and *ZFHX3* (rs7199343, *p*_combined_ = 2.37×10^−6^) most significantly associated. Sixteen associated variants with a minor allele frequency of >0.05 that lay within or close to known genes were fine-mapped with HapMap tagging SNPs in 781 KD cases, including 590 from the discovery and replication stages. Original or tagging SNPs in eight of these genes replicated the original findings, with seven genes having further significant markers in adjacent regions. In four genes (*ZFHX3*, *NAALADL2*, *PPP1R14C*, and *TCP1*), the neighboring markers were more significantly associated than the originally associated variants. Investigation of functional relationships between the eight fine-mapped genes using Ingenuity Pathway Analysis identified a single functional network (*p* = 10^−13^) containing five fine-mapped genes—*LNX1*, *CAMK2D*, *ZFHX3*, *CSMD1*, and *TCP1*—with functional relationships potentially related to inflammation, apoptosis, and cardiovascular pathology. Pair-wise blood transcript levels were measured during acute and convalescent KD for all fine-mapped genes, revealing a consistent trend of significantly reduced transcript levels prior to treatment. This is one of the first GWAS in an infectious disease. We have identified novel, plausible, and functionally related variants associated with KD susceptibility that may also be relevant to other cardiovascular diseases.

## Author Summary

Kawasaki disease is an inflammatory pediatric condition that damages the coronary arteries in a quarter of untreated patients and is the commonest cause of childhood acquired heart disease in developed countries. While the infectious trigger(s) remain unknown, epidemiologic evidence suggests that human genetic variation underlies the susceptibility. In order to identify novel mechanisms that may predispose to this disease, we undertook a genome-wide association study, which investigates genetic determinants without prior supposition regarding the loci of interest. This was amongst the first complex infectious diseases to be studied in this way and one of the largest genetic studies of Kawasaki disease with 893 cases. We identified and confirmed 40 SNPs and six haplotypes, identifying 31 genes, in an international cohort of Caucasian patients. We followed up 16 SNPs where the associated genetic variant was more common and was situated within a gene, confirming eight SNPs by fine-mapping across the entire gene. Of these eight genes, seven were expressed in blood and five showed significantly different gene expression in paired patient samples taken during acute and convalescent Kawasaki disease. Five of the eight genes also appear to be involved in a single putative functional network of interacting genes. These novel genes and pathways may ultimately lead to novel diagnostics and treatment for Kawasaki disease.

## Introduction

Kawasaki disease (KD; MIM 611775) is an inflammatory vasculitis predominantly affecting young children [1]. It is characterized by a striking propensity for coronary artery damage, which occurs in approximately 25% of untreated and 3–5% of treated children. KD is the commonest cause of heart disease acquired in childhood in developed nations and in those who manifest coronary artery damage, KD may be associated with serious cardiovascular sequelae in adulthood [2]. The long-term cardiovascular implications of KD in those without overt coronary artery lesions are unclear. The etiology of KD is unknown, but it is thought to reflect an abnormal and sustained inflammatory response to one or more infectious triggers in genetically susceptible individuals [1],[3]. No consistent etiologic agent for KD has been identified, hampering accurate and timely diagnosis and the development of optimal management strategies.

The incidence of KD varies markedly in different ethnic groups, with the highest incidence in North East Asian populations. KD affects approximately 1 in 150 Japanese children [1] and is responsible for 1–2% of all pediatric hospital admissions in South Korea [4]. There are strong epidemiologic data to support a substantial genetic contribution to KD susceptibility. The Japanese incidence (135–200/100 000<5 years of age) is 10–15 times greater than the Caucasian incidence (9–17/100 000<5 years of age) [1] and this difference is maintained in American children of Japanese descent resident in the US [5]. Other Asian populations in the UK [6],[7] and the US [8] also have a significantly higher incidence than non-Asians residing in the same geographic location. The familial inheritance pattern of KD is in keeping with a polygenic complex disease and in multi-case pedigrees, KD occurs in family members at different times and geographic locations [9]. Across all populations KD is approximately 1.6 times more common in males [3]. The sibling risk ratio for KD in the Japanese is approximately 10 during an epidemic [10] and 6 overall [11]. KD is over twice as common in the children of parents who themselves had KD in childhood, with multi-generational pedigrees often having more than one child affected, an earlier age of onset and a more severe phenotype [11].

A genome-wide linkage study identified three regions exhibiting modest linkage in Japanese KD sibling pairs [12]. Detailed association analysis of the linkage peak at chromosome 19q13.2 identified a significantly associated functional variant in *ITPKC* (Gene ID:80271), a negative regulator of T cell activation [13]. In both Japanese and US Caucasian children with KD, this variant was associated with an approximate doubling of KD risk [13]. Other investigations of KD susceptibility determinants to date have been candidate gene association studies. A number of immunologic and cardiovascular-related loci have revealed genetic associations of modest effect size, but studies have often been under-powered and the findings have rarely been replicated in independent populations. As these associated candidate loci are likely to account for only a small proportion of the overall genetic susceptibility to KD, we undertook a genome-wide association study (GWAS) to identify novel loci that might mediate susceptibility to KD. We performed the initial GWAS in a well-characterized Dutch Caucasian population and tested the most significantly associated SNPs and haplotypes in a large independent cohort of predominantly Caucasian trios from three countries. Fine-mapping of sixteen variants with a minor allele frequency (MAF) of >0.05 that lay within or close to known genes identified eight significantly associated variants, five of which may be functionally related.

## Results

### Genome-Wide Association Study

**Figure 1:**
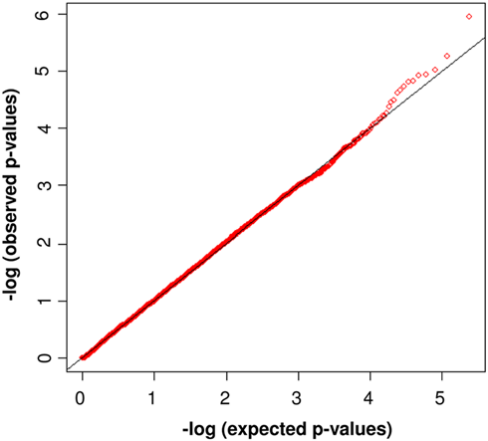
Quantile-Quantile Plot of Allelic Distributions. Allelic association analysis of expected *versus* observed P-values of 223,922 SNPs in 107 KD cases and 134 controls. Red dots showing deviations from the line of equality indicate either that the theoretical distribution was incorrect, or that the sample was contaminated with values generated by a true association.

We included 119 KD cases and 135 controls in the initial GWAS (Table 1). Ten non-Caucasian subjects were excluded following admixture analysis by Eigenstrat [14]. Three samples were excluded due to genotyping call rates <93%. The final GWA analysis therefore consisted of 107 KD subjects and 134 controls. Of the 262,264 SNPs on the Affymetrix 250 K NSP chip, 18,211 had a call rate of <93%, 18,981 were monomorphic (MAF<0.1%) and 1,150 deviated significantly from HWE in the control group. 223,922 SNPs were therefore available for analysis, of which 5,571 were on the X chromosome. A total of 14,065 SNPs were significantly associated (p<0.05). The quantile-quantile plot between observed and expected allele frequencies showed deviation from expected with p<∼10^−4^, suggesting the presence of true associations [15] (Figure 1).

**Table 1:** Demographic and Clinical Characteristics of Subjects in the GWAS (Dutch Case-Control Samples) and Replication Study (Australian, US And UK Families).

Characteristics	Genome-Wide Association Cohort	Family-based Follow-up Cohort
Cases (% male)	119 (58.8)	583 (63.1)
Controls (% male)	135 (63.8)	1357 (48.8) a
Caucasian ethnicity (%)	241/254 (94.5)	-
Coronary artery abnormality^b^ (%)	27/119 (22.7)	155/583 (26.5)

a parents and healthy siblings of proband.

b significant coronary dilatation on any echocardiogram during the KD illness.

### Follow-Up Replication Study in Independent Cohorts

We undertook a replication study in an independent cohort of KD cases and parental controls, using an exact replication strategy, genotyping only the most significantly associated variants by a different genotyping technology. After verifying family relationships and checking sample duplications, 63 samples were excluded from further analysis. Thus 1,903 members of 583 KD families, including 498 trios, were tested in the follow-up association analysis.

### Replication of Individual SNP Associations

**Figure 2:**
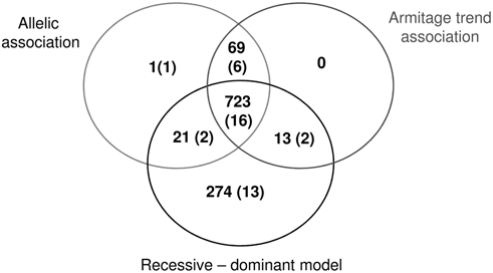
Selection and Verification of SNPs And Haplotypes from the GWAS Analysis. GWAS SNPs and haplotypes were ranked by P-value and the top 1101 (of 14,065 total associated) SNPs and 35 (of 3,549 non-overlapping total associated) haplotypes were carried through to the follow-up stage. Numbers in parenthesis indicate the number of variants selected from the GWAS by each method of selection that were subsequently replicated in the family-based study.

The 1,148 most significantly associated SNPs variants (corresponding to a minimum significance level of p<0.0024) were selected by a combination of Armitage trend test, recessive-dominant and allelic association analysis (Figure 2). SNPs were genotyped in the follow-up cohort by a custom Illumina Oligo Pool Assay, in which 1,116 SNPs were successfully genotyped (Table S1). Fifteen SNPs failed quality filters, leaving a total of 1,101 SNPs.

Significant associations with KD susceptibility were replicated for 40 SNPs (Table 2). Twenty eight lay either in or within 50 kb of known genes. The most highly associated SNP was located in the gene for N-acetylated alpha-linked acidic dipeptidase-like 2 (*NAALADL2*, Gene ID:254827), (combined OR from case-control and family analyses = 1.43 (1.32–1.53); p_combined_ = 1.13×10^−6^). Three SNPs were located in introns of the gene for AT-binding transcription factor 1 (*ZFHX3*, Gene ID:463), all of which had a protective effect (OR_combined_ = 0.64 (0.52–0.75), 0.68 (0.57–0.79), 0.73 (0.62–0.84); p_combined_ = 2.37×10^−6^, 7.06×10^−5^, 5.35×10^−4^, respectively). Two of these SNPs, rs7199343 and rs10852516, were only 647 bp apart and in high linkage disequilibrium (LD) (r^2^ = 0.93), whereas the third SNP, rs11075953, was ∼10 kb distant and in less LD (r^2^ = 0.46). Two SNPs, rs1010824 and rs6469101, which were 325 bp apart and in complete LD (r^2^ = 1), were situated ∼10 kb 3′ of the angiopoietin 1 gene (*ANGPT1*, Gene ID:284) (OR_combined_ = 0.58 (0.43–0.73) and 0.6 (0.45–0.76); p_combined_ = 3.39×10^−5^ and 5.44×10^−5^, respectively).

**Table 2:** Significantly Associated Variants Confirmed in the Replication Study.

SNP analysis										
Marker	Chr.	MAF	Genes^a^	Genome-Wide Association		Family-based Follow-up study			Combined values	
				p value	OR (95%CI)	p value	OR (95%CI)^b^	Info.fam^c^	p value	OR (95%CI)
rs3842967	1	0.17	*MARK1*	2.69*10^−3^	0.55 (0.34–0.8)	4.68*10^−2^	0.8 (0.58–1.1)	278	1.26*10^−3^	0.70 (0.58–0.83)
rs10183521	2	0.46	*-*	4.15*10^−4^	1.37 (0.94–1.99)	1.79*10^−2^	1.24 (0.97–1.58)	366	9.50*10^−5^	1.28 (1.17–1.39)
rs6432366	2	0.36	*-*	1.05*10^−3^	1.81 (1.28–2.7)	1.03*10^−2^	1.28 (0.98–1.66)	337	1.35*10^−4^	1.44 (1.33–1.55)
rs10519011	2	0.008	*CEP68*	2.39*10^−3^	10.5(1.3–85.2)	3.08*10^−2^	2 (0.87–4.57)	40	7.73*10^−4^	2.58 (2.16–3.01)
rs10153799	2	0.02	*-*	1.52*10^−3^	4 (1.6–9.9)	4.23*10^−2^	1.92 (0.83–4.45)	40	6.84*10^−4^	3.09 (2.76–3.42)
rs9834548^d^	3	0.4	*-*	2.72*10^−4^	0.5 (0.34–0.73)	2.35*10^−3^	0.73 (0.55–0.97)	343	9.77*10^−6^	0.64 (0.53–0.75)
rs358056	3	0.25	*CACNA2D3*	6.14*10^−4^	1.92 (1.22–3)	2.82*10^−2^	1.26 (0.94–1.69)	281	2.07*10^−4^	1.43 (1.31–1.56)
rs16849640	3	0.1	*CLSTN2*	4.48*10^−4^	0.26 (0.11–0.62)	4.46*10^−2^	0.8 (0.5–1.28)	142	2.36*10^−4^	0.62 (0.41–0.82)
rs17531088	3	0.47	*NAALADL2*	3.39*10^−4^	1.46(1.0–2.1)	1.64*10^−4^	1.41 (1.1–1.8)	375	1.13*10^−6^	1.43 (1.32–1.53)
rs937130	3	0.48	*GABRR3*	1.42*10^−3^	1.66 (1.16–2.39)	3.81*10^−2^	1.19 (0.93–1.53)	373	5.87*10^−4^	1.33 (1.22–1.43)
rs17531554	4	0.22	*CAMK2D*	1.34*10^−3^	2.1(1.3–3.3)	3.27*10^−2^	1.29 (0.93–1.77)	246	4.82*10^−4^	1.52 (1.38–1.65)
rs16869942	4	0.008	*KCNIP4*	1.19*10^−3^	8 (1.77–36.34)	8.15*10^−3^	2 (1.01–3.93)	63	1.22*10^−4^	2.62 (2.28–2.96)
rs4864471	4	0.18	*LNX1,LOC441016*	6.05*10^−4^	2.47 (1.3–4.5)	4.01*10^−3^	1.5 (1.03–2.19)	186	3.38*10^−5^	1.74 (1.57–1.91)
rs4333331	5	0.39	*PDZD2*	8.59*10^−4^	0.82 (0.56–1.18)	2.78*10^−2^	0.81 (0.62–1.06)	360	2.78*10^−4^	0.81 (0.71–0.92)
rs7739088	6	0.27	*C6orf70, C6orf122*	1.51*10^−3^	0.49 (0.31–0.77)	1.77*10^−2^	0.77 (0.56–1.05)	303	3.09*10^−4^	0.67 (0.54–0.80)
rs7755143	6	0.12	*PI16,MTCH1*	1.07*10^−3^	2.25 (1.37–3.68)	4.42*10^−2^	1.27 (0.92–1.77)	229	5.17*10^−4^	1.53 (1.38–1.67)
rs1408505	6	0.08	*PPP1R14C*	2.15*10^−3^	0.51 (0.33–0.79)	4.29*10^−2^	0.79 (0.57–1.09)	263	9.46*10^−4^	0.68 (0.55–0.81)
rs2273828	6	0.48	*TCP1*	6.35*10^−4^	0.6 (0.4–0.87)	4.15*10^−2^	0.82 (0.63–1.07)	355	3.04*10^−4^	0.74 (0.63–0.85)
rs9392158	6	0.11	*RIOK1*	7.89*10^−4^	2.4 (1.38–4.26)	8.30*10^−3^	1.48 (0.99–2.19)	172	8.47*10^−5^	1.75 (1.58–1.92)
rs17157642	7	0.18	*-*	1.30*10^−3^	2.37 (1.38–4.0)	4.26*10^−2^	1.28 (0.92–1.79)	225	5.99*10^−4^	1.53 (1.38–1.68)
rs196751	7	0.57	*DGKB*	1.34*10^−3^	0.55 (0.38–0.89)	4.99*10^−2^	0.83 (0.64–1.08)	362	7.12*10^−4^	0.73 (0.62–0.83)
rs17167055	7	0.04	*COL28A1*	1.84*10^−3^	5.02 (1.64–15.37)	2.33*10^−2^	1.61 (0.92–2.79)	88	4.73*10^−4^	2.06 (1.79–2.33)
rs10046555	7	0.14	*TNS3*	1.78*10^−3^	0.52 (0.31–0.86)	4.66*10^−2^	0.78 (0.55–1.11)	242	8.61*10^−4^	0.69 (0.54–0.83)
rs1010824	8	0.17	*ANGPT1*	4.08*10^−4^	0.38 (0.22–0.66)	5.96*10^−3^	0.69 (0.47–1)	228	3.39*105^4^	0.58 (0.43–0.73)
rs6469101	8	0.17	*ANGPT1*	1.12*10^−3^	0.39 (0.21–0.73)	3.62*10^−3^	0.69 (0.48–1)	230	5.44*10^−5^	0.60 (0.45–0.76)
rs2912272	8	0.14	*CSMD1*	2.52*10^−3^	2.54 (1.36–4.74)	4.60*10^−2^	1.37 (0.89–2.11)	144	1.17*10^−3^	1.69 (1.51–1.88)
rs328879	9	0.35	*-*	1.10*10^−3^	0.53 (0.36–0.79)	7.27*10^−4^	0.72 (0.55–0.95)	351	1.20*10^−5^	0.65 (0.54–0.77)
rs10984642	9	0.15	*-*	4.27*10^−5^	0.27 (0.14–0.52)	4.28*10^−2^	0.75 (0.5–1.12)	190	2.60*10^−5^	0.57 (0.4–0.74)
rs16915269	9	0.12	*-*	7.84*10^−4^	3.09 (1.55–6.14)	5.51*10^−3^	1.56 (1.02–2.39)	147	5.77*10^−5^	1.92 (1.72–2.11)
rs11063543	12	0.11	*-*	1.19*10^−3^	2.5 (1.42–4.44)	4.19*10^−2^	1.28 (0.92–1.79)	231	5.43*10^−4^	1.53 (1.38–1.68)
rs10862477	12	0.31	*ZDHHC17*	2.55*10^−3^	1.33 (0.91–1.95)	4.97*10^−2^	1.20 (0.93–1.54)	354	1.27*10^−3^	1.24 (1.13–1.35)
rs285032	13	0.51	*FARP1*	3.15*10^−4^	0.52 (0.36–0.75)	3.71*10^−3^	0.77 (0.6–0.99)	381	1.71*10^−5^	0.68 (0.58–0.79)
rs7151781	14	0.41	*-*	1.70*10^−3^	1.58 (1.09–2.29)	1.09*10^−2^	1.29 (1–1.68)	332	2.20*10^−4^	1.38 (1.27–1.49)
rs2899383	15	0.38	*-*	2.00*10^−3^	0.71 (0.49–1.04)	4.37*10^−2^	0.83 (0.64–1.08)	354	9.06*10^−4^	0.79 (0.68–0.90)
rs4775110	15	0.26	*RNF111*	1.91*10^−3^	1.8 (1.2–2.71)	1.14*10^−2^	1.26 (0.98–1.63)	353	2.56*10^−4^	1.40 (1.29–1.51)
rs7199343	16	0.32	*ZFHX3*	8.73*10^−5^	0.48(0.32–0.71)	1.16*10^−3^	0.73 (0.55–0.97)	332	2.37*10^−6^	0.64 (0.52–0.75)
rs10852516	16	0.66	*ZFHX3*	2.33*10^−3^	0.55 (0.37–0.81)	2.31*10^−3^	0.75 (0.57–0.98)	354	7.06*10^−5^	0.68 (0.57–0.79)
rs11075953	16	0.4	*ZFHX3*	2.27*10^−3^	0.59 (0.4–0.87)	2.16*10^−2^	0.8 (0.61–1.05)	349	5.35*10^−4^	0.73 (0.62–0.84)
rs8059315	16	0.14	*GLG1*	1.38*10^−4^	1.4 (0.87–2.26)	4.74*10^−2^	1.27 (0.91–1.75)	241	8.45*10^−5^	1.31 (1.17–1.45)
rs7208206	17	0.02	*-*	2.15*10^−3^	0.23 (0.08–0.66)	3.50*10^−2^	0.58 (0.27–1.24)	65	8.89*10^−4^	0.43 (0.13–0.73)
**Haplotype analysis**										
rs17348835, rs17348849	2	-	-	2.51*10^−3^	1.85 (1.25–2.73)	2.22*10^−2^	1.50 (1.05–2.13)	206	5.99*10^−4^	1.64 (1.27–2.13)
rs7558220, rs357752	2	*-*	*MPHOSPH10*	4.90*10^−3^	0.38 (0.19–0.75)	7.10*10^−3^	0.40 (0.20–0.80)	114	3.91*10^−4^	0.39 (0.24–0.63)
rs7558220, rs357752, rs1458868, rs357729, rs11674899	2	*-*	*MPHOSPH10*	1.68*10^−2^	3.61 (1.22–7.46)	2.50*10^−2^	3.42 (1.09–10.7)	78	3.69*10^−3^	3.52 (1.60–7.75)
rs7572970, rs716615, rs12692592	2	*-*	*RBMS1*	1.37*10^−3^	0.67 (0.53–0.86)	3.60*10^−3^	0.71 (0.56–0.89)	297	6.51*10^−5^	0.69 (0.58–0.82)
rs224666, rs10767911, rs224618290	11	-	-	1.80*10^−2^	0.43 (0.22–0.87)	3.50*10^−2^	0.45 (0.21–0.97)	87	5.27*10^−3^	0.44 (0.26–0.74)
rs6034368, rs202479	20	*-*	*LOC441938*	9.32*10^−3^	0.73 (0.57–0.92)	2.00*10^−2^	0.75 (0.59–0.96)	290	1.79*10^−3^	0.74 (0.62–0.88)

a Genes containing the SNP or the closest gene up to 50 kb up and downstream of the SNP.

b Odds ratio for haplotype TDT was calculated based on haplotypes reconstructed from FBAT software viewhap command.

c Number of informative families.

d build35 rs11711042.

### Replication of Haplotypic Associations

166 SNPs within the most highly associated thirty five haplotype blocks (corresponding to p<2×10^−3^) were genotyped using a Sequenom iPLEX platform in the replication cohort (Table S2). Twenty-nine SNPs could not be genotyped, leaving 137 SNPs for further analysis. Significant associations were verified for six haplotypes, of which four were within known genes. Two haplotypes lay within the gene encoding M-phase phosphoprotein 10 (U3 small nucleolar ribonucleoprotein), *MPHOSPH10* (Gene ID:10199). One of these haplotypes was associated with increased susceptibility (OR_combined_ = 3.52 (1.6–7.75); p = 3.69×10^−3^) and the other with protection from KD (OR_combined_ = 0.39 (0.24–0.63); p = 3.91×10^−4^) (Table 2).

### Fine-Mapping of More Common Associated Variants Lying within or Close to Known Genes

**Figure 3:**
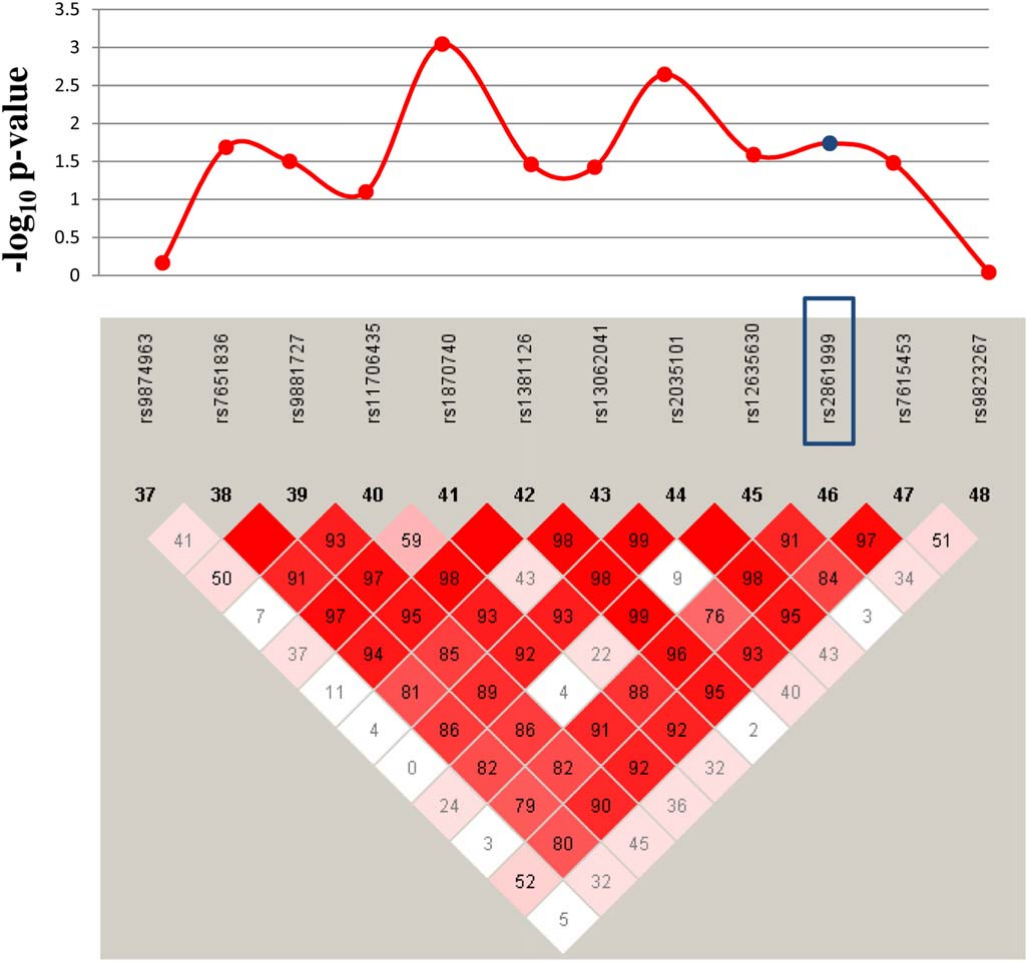
Linkage Disequilibrium Plot of a Region of *NAALADL2* Containing the Most Significantly Associated Variants. The upper portion shows -log (p-values) of tagging SNPs (r^2^>0.8) of the polymorphisms, initially identified in our GWAS (rs2861999) and replication study (rs17531088), which are highlighted in blue. D′ values are shown inside each diamond. Red diamonds without a number represent D′ = 1.

In order to narrow the region of association within selected genes, we carried out fine-mapping of sixteen of the genes replicated in the family study (Table S3). We fine-mapped genes identified by variants with a MAF of >0.05 lying within 5 kb of known genes. Polymorphisms from eight of the sixteen fine-mapped genes showed significant combined p-values (Table 3). In three genes the tagging SNP showed the most significant genetic association; *LNX1* (Gene ID:84708) (rs7660884, p_combined_ = 1.8×10^−3^), *CAMK2D* (Gene ID:817) (rs11728021, p_combined_ = 1.3×10^−2^) and *CSMD1* (Gene ID:64478) (rs2912272, p_combined_ = 3.5×10^−2^), indicating that the initial GWAS probably identified a disease-associated haplotype. Conversely within *NAALADL2*, the most significantly associated gene identified in the replication study, a polymorphism (rs1870740) located 23 kb away from the tagging SNP (rs2861999) was the most significantly associated (p_combined_ = 9×10^−4^). A linkage disequilibrium plot of this region indicated that these two SNPs belong to distinct haplotype blocks (Figure 3). The transcription factor *ZFHX3* had a number of SNPs with more significant associations than the variant tagging the replicated polymorphism (Figure S2). Linkage disequilibrium plots of all fine-mapped genes are presented in the online supplemental material (Figure S3, S4, S5, S6, S7, and S8).

**Table 3:** Combined P-Values of Fine-Mapped Genes.

			Case-control		Family-based					
Marker	Chr	Gene	Allelic p value	ORs(95%CI)	p value	ORs(95%CI)^a^	Info.fam^b^	Combined p value	Location	Consequence
rs6801807	3	*NAALADL2*	0.51	0.89(0.63–1.25)	3.8*10^−3^	0.77(0.64–0.93)	275	1.3*10^−2^	176150140	UPSTREAM
rs9813316	3	*NAALADL2*	0.31	0.81(0.55–1.2)	1.8*10^−2^	0.77(0.63–0.94)	234	3.4*10^−2^	176191353	UPSTREAM
rs6806492	3	*NAALADL2*	0.36	0.83(0.56–1.2)	1.9*10^−3^	0.70(0.57–0.87)	226	5.6*10^−3^	176193394	UPSTREAM
rs7651836	3	*NAALADL2*	0.27	0.83(0.59–1.15)	1*10^−2^	0.88(0.73–1.06)	289	2*10^−2^	176344378	INTRONIC
rs9881727	3	*NAALADL2*	0.51	0.90(0.66–1.21)	9.8*10^−3^	0.85(0.71–1.02)	325	3.1*10^−2^	176344679	INTRONIC
rs1870740	3	*NAALADL2*	0.38	0.88(0.65–1.17)	2.2*10^−4^	0.75(0.64–0.89)	341	9*10^−4^	176364140	INTRONIC
rs1381126	3	*NAALADL2*	0.07	0.66(0.42–1.02)	0.07	0.74(0.58–0.96)	187	3.4*10^−2^	176369549	INTRONIC
rs13062041	3	*NAALADL2*	0.51	0.90(0.68–1.21)	1.2*10^−2^	0.83(0.70–0.99)	337	3.7*10^−2^	176377638	INTRONIC
rs2035101	3	*NAALADL2*	0.65	1.07(0.8–1.44)	3.7*10^−4^	1.31(1.11–1.57)	330	2.2*10^−3^	176378534	INTRONIC
rs2861999^c^	3	*NAALADL2*	0.31	1.17(0.87–1.56)	8.3*10^−3^	1.23(1.04–1.46)	335	1.8*10^−3^	176387101	INTRONIC
rs7615453	3	*NAALADL2*	0.3	0.84(0.62–1.15)	1.7*10^−2^	0.83(0.70–1.00)	313	3.3*10^−2^	176392646	INTRONIC
rs6788338	3	*NAALADL2*	3.5*10^−2^	0.72(0.53–0.97)	0.07	0.95(0.80–1.13)	311	1.8*10^−2^	176538191	INTRONIC
rs17082951	4	*LNX1*	0.98	1.00(0.73–1.39)	7*10^−3^	1.24(1.03–1.50)	304	4.1*10^−2^	54087872	INTRONIC
rs6554112	4	*LNX1*	1.1*10^−2^	1.50(1.10–2.03)	0.5	1.04(0.87–1.25)	296	3.6*10^−2^	54113392	INTRONIC
rs7660884^c^	4	*LNX1*	1.1*10^−2^	1.77(1.15–2.74)	1.7*10^−2^	1.51(1.14–2.00)	152	1.8*10^−3^	54121365	INTRONIC
rs4834340	4	*CAMK2D*	0.24	0.84(0.62–1.12)	1.7*10^−2^	0.86(0.72–1.02)	297	2.7*10^−2^	114620118	INTRONIC
rs11728021^c^	4	*CAMK2D*	0.28	1.23(0.85–1.76)	6.6*10^−3^	1.23(0.98–1.52)	232	1.3*10^−2^	114698102	INTRONIC
rs1408504	6	*PPP1R14C*	4.8*10^−2^	0.70(0.55–0.99)	1.1*10^−2^	0.86(0.73–1.02)	337	4.4*10^−3^	150513287	INTRONIC
rs12332856^c^	6	*PPP1R14C*	1.3*10^−2^	0.64(0.45–0.9)	0.22	0.88(0.72–1.08)	242	2*10^−2^	150514181	INTRONIC
rs2144400	6	*PPP1R14C*	0.42	0.86(0.59–1.23)	5.3*10^−3^	0.79(0.63–0.98)	222	1.5*10^−2^	150547398	INTRONIC
rs2295899^c^	6	*TCP1*	0.09	0.77(0.58–1.03)	0.09	0.85(0.72–1.01)	315	4.7*10^−2^	160122157	INTRONIC
rs3818298	6	*TCP1*	0.23	0.78(0.52–1.15)	1.9*10^−2^	0.75(0.60–0.92)	244	2.8*10^−2^	160126706	INTRONIC
rs10215596	7	*DGKB*	0.18	0.69(0.4–1.17)	2.5*10^−2^	0.82(0.61–1.11)	133	2.9*10^−2^	14326534	INTRONIC
rs12699612	7	*DGKB*	0.69	1.07(0.76–1.51)	2*10^−3^	0.8(0.66–0.96)	299	1.1*10^−2^	14388440	INTRONIC
rs196751^d^	7	*DGKB*	2.5*10^−2^	0.71(0.53–0.95)	0.1	0.88(0.74–1.03)	311	1.8*10^−2^	14443632	INTRONIC
rs1431518	7	*DGKB*	0.08	0.62(0.36–1.06)	0.06	0.72(0.53–0.99)	113	3.4*10^−2^	14458033	INTRONIC
rs1367782	7	*DGKB*	0.06	0.75(0.56–1.01)	0.12	0.87(0.73–1.02)	322	4.6*10^−2^	14560761	INTRONIC
rs1431513	7	*DGKB*	8.2*10^−3^	1.50(1.12–2.00)	0.84	1.03(0.87–1.22)	310	4.1*10^−2^	14571423	INTRONIC
rs6949687	7	*DGKB*	9*10^−3^	0.67(0.5–0.9)	0.21	0.88(0.74–1.04)	329	1.4*10^−2^	14577139	INTRONIC
rs1404611	7	*DGKB*	3.2*10^−2^	0.63(0.42–0.95)	0.12	0.83(0.65–1.06)	176	2.5*10^−2^	14638173	INTRONIC
rs2912272^d^	8	*CSMD1*	9.7*10^−2^	1.55(0.93–2.57)	0.06	1.5(1.10–2.06)	124	3.5*10^−2^	3885737	UPSTREAM
rs8055870	16	*ZFHX3*	0.11	0.78(0.58–1.05)	0.05	0.9(0.76–1.07)	347	3.7*10^−2^	71529591	INTRONIC
rs7193297	16	*ZFHX3*	1.2*10^−2^	0.68(0.51–0.91)	0.35	1.00(0.84–1.19)	311	3.7*10^−2^	71551332	Ser72Ala
rs4788683	16	*ZFHX3*	7.2*10^−4^	0.59(0.44–0.79)	0.31	0.96(0.80–1.14)	312	2.1*10^−3^	71555248	INTRONIC
rs11075953^c^	16	*ZFHX3*	1.2*10^−2^	0.67(0.49–0.9)	1.1*10^−2^	0.82(0.68–0.97)	299	1.3*10^−3^	71557266	INTRONIC
rs9921395	16	*ZFHX3*	0.07	0.76(0.56–1.02)	8.4*10^−4^	0.77(0.65–0.91)	319	6.8*10^−4^	71559458	INTRONIC
rs9937546	16	*ZFHX3*	0.07	0.75(0.55–1.02)	2*10^−4^	0.72(0.60–0.87)	284	1.9*10^−4^	71561220	INTRONIC
rs12445917	16	*ZFHX3*	7.8*10^−3^	0.61(0.42–0.86)	7.8*10^−3^	0.76(0.61–0.94)	226	6.6*10^−4^	71562038	INTRONIC
rs11075954	16	*ZFHX3*	0.95	1.00(0.74–1.32)	3.6*10^−3^	1.26(1.06–1.49)	310	2.3*10^−2^	71569665	INTRONIC
rs11640395	16	*ZFHX3*	6.8*10^−3^	0.61(0.42–0.86)	3.3*10^−2^	0.82(0.67–1.02)	225	2.1*10^−3^	71572247	INTRONIC
rs17681554	16	*ZFHX3*	2.2*10^−2^	0.70(0.52–0.94)	1*10^−3^	0.78(0.66–0.92)	317	2.7*10^−4^	71574269	INTRONIC
rs17692597^c^	16	*ZFHX3*	1.3*10^−2^	0.67(0.49–0.91)	1.2*10^−2^	0.86(0.71–1.03)	290	1.5*10^−3^	71579774	INTRONIC
rs7193343	16	*ZFHX3*	1.5*10^−3^	0.52(0.36–0.78)	0.11	0.8(0.65–1.00)	222	1.5*10^−3^	71586661	INTRONIC
rs11075958	16	*ZFHX3*	1.3*10^−2^	0.63(0.44–0.9)	0.08	0.87(0.70–1.08)	204	8.5*10^−3^	71591370	INTRONIC
rs719353	16	*ZFHX3*	2.8*10^−3^	0.63(0.47–0.85)	0.38	0.90(0.76–1.06)	331	8.5*10^−3^	71600052	INTRONIC
rs4788694	16	*ZFHX3*	5.9*10^−3^	0.66(0.5–0.88)	0.88	0.96(.082–1.14)	313	3.2*10^−2^	71627584	INTRONIC

a Haplotype relative risk odds ratio.

b Number of informative families.

c tagging SNP.

d same SNP typed (from GWAS and follow up).

### Allele Frequencies in Different Ethnic Groups and Identification of the Ancestral Allele

In order to investigate differences in allele frequencies of associated SNPs between Japanese, where the incidence of KD is approximately twenty times higher than that of Caucasians, we compared MAF data for associated variants using HapMap. In addition we investigated the ancestral allele of each associated SNP from available data from higher primates (Chimpanzee and Macaque) (Table S4). All (but one) associated alleles of SNPs within *ZFHX3* had higher frequencies in the Japanese population. The associated allele (T) from the most significantly associated SNP (rs9937546 p_combined_ = 1.9×10^−4^), had an allele frequency of 0.922 in the Japanese, compared to 0.633 in Caucasians. Despite the high frequencies in human populations, the rs9937546 T allele was not the ancestral allele, which might indicate rapid fixation in the population due to an unidentified evolutionary advantage. In contrast the associated allele of rs2912272 from *CSMD1*, was absent in Japanese populations, which could indicate genetic heterogeneity in this gene, with other variants associated with susceptibility in the Japanese. In *CSMD1* the ancestral allele is the major allele in humans.

### Gene Network Analysis

**Figure 4:**
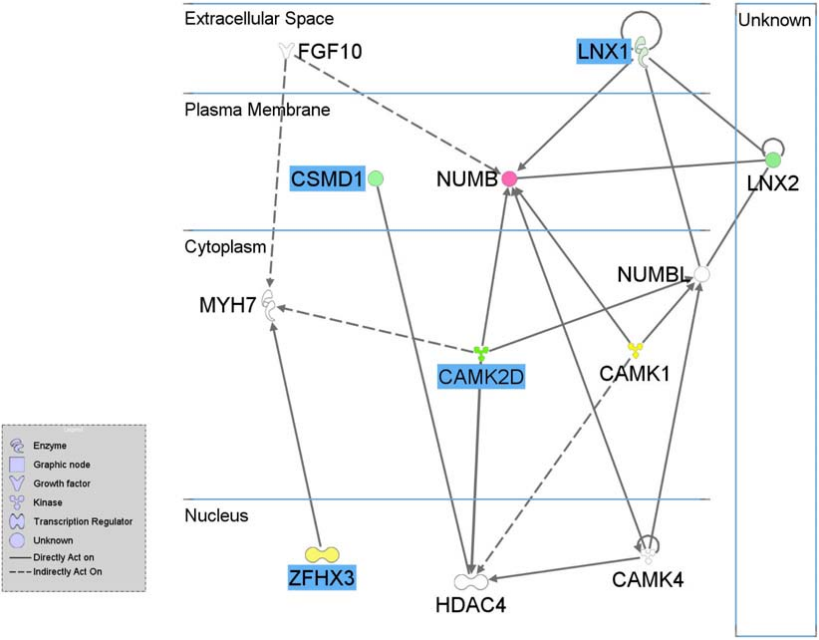
Putative Gene Network Derived from Ingenuity Pathway Analysis Software. Edges are displayed with labels that describe the nature of the relationship between the nodes. The lines between genes represent known interactions, with solid lines representing direct interactions and dashed lines representing indirect interactions. Nodes are displayed using various shapes that represent the functional class of the gene product (see legend). Blue highlighting indicates GWAS associated genes and non-highlighted genes are those identified by IPA. Genes which showed differential transcript levels in acute versus convalescent KD are colored red if the level was significantly higher during acute KD, green if the level was significantly lower and yellow if there was no significant change, with color intensity related to fold change. Genes not colored were not measured.

We explored possible functional relationships between the eight genes confirmed by fine-mapping using the *Ingenuity Pathway Analysis* (IPA) *Knowledge Base*. Unsupervised IPA network analysis identified a single cluster of 35 genes that included five of the eight associated genes and 26 additional genes, that was unlikely to occur by chance (p = 10^−13^). Highlights from the group are shown in Figure 4, concentrating on the close connection between four of the associated fine-mapped genes and eight IPA identified genes that create a plausible biological network.

### Gene Expression

We investigated the whole blood transcript levels of the eight fine-mapped associated genes, and three of the IPA identified genes, using TaqMan quantitative PCR, in 27 patients with paired samples from their acute KD stage (prior to treatment) and their convalescence, using the transcript levels of the gene 18S as a loading control. The blood cell profiles of these patients have been described previously [16]. Of the 54 RNA samples, two pairs were excluded because of inadequate RNA quality.

Of the eight GWAS identified genes, five showed significantly lower abundance at the acute phase compared to convalescence, while two showed no change and expression of one was not detected in peripheral blood (Table 4). For the three IPA identified genes investigated, one showed significantly higher abundance, one showed significantly lower abundance and one showed no significant difference between acute and convalescence samples (Table 4). *NAALADL2* (NM_207015) showed the largest fold change (FC) (FC = −6.3, p = 1.55×10^−4^), while *CAMK2D* (NM_001221) showed the most significant difference (FC = −2.5, p = 1.53×10^−7^) (Table 4). Relative transcript abundance is represented in the network diagram (Figure 4), with red indicating greater abundance in acute samples compared to convalescent samples, green lower abundance and yellow no change.

**Table 4:** Differential Gene Transcript Levels in Acute *versus* Convalescent KD.

Gene Name	Transcript ID	Source^a^	Acute KD median RQ (S.D)	Convalescent KD median RQ (S.D)	Differential transcript levels	Fold Change
*CAMK2D*	NM_001221	GWAS	1.97(1.64)	5.00(1.89)	1.530E-07	−2.5
*CSMD1*	NM_015336	GWAS	0.11(0.06)	0.20(0.39)	1.20E-03	−1.8
*DGKB*	NM_145695	GWAS	Not detected	Not detected	-	-
*LNX1*	NM_032622	GWAS	0.10(0.58)	0.17(10.07)	3.05E-02	−1.7
*NAALADL2*	NM_207015	GWAS	0.16(0.67)	1.00(1.75)	1.55E-04	−6.3
*PPP1R14C*	NM_030949	GWAS	1.52(2.72)	1.94(3.15)	-	-
*TCP1*	NM_030752	GWAS	1.34(1.13)	2.41(1.26)	9.86E-03	−1.8
*ZFHX3*	NM_006885	GWAS	1.20(0.72)	1.61 (2.9)	-	-
*CAMK1D*	NM_153498	I	1.07(0.53)	1.37(0.46)	-	-
*LNX2*	NM_153371	I	1.38(0.66)	1.92(0.69)	7.35E-04	−1.4
*NUMB*	NM_001005743	I	0.64(0.31)	0.29(0.12)	9.82E-05	2.2
*18s*	Endogenous Control	C	Ct = 13.64(0.19)	Ct = 13.55(0.12)	-	-

a GWAS = genome wide association study identified genes that were successfully fine-mapped.

I = genes identified by Ingenuity Pathway Analysis; C = control; - = Not significant.

Transcript levels of eight fine-mapped genes and three genes suggested by Ingenuity Pathway Analysis (I) were compared to transcript abundance of the loading endogenous control gene 18S, (C) in paired acute (prior to IVIg treatment) and convalescent KD samples from the same individuals (n = 25). Numbers are median (S.D.) relative values compared to endogenous control and fold change (FC) calculated by median acute levels divided by median convalescent levels. For the 18S control, numbers represent the mean (S.D.) PCR cycle at detection (Ct). Significance of differential transcript levels was determined using the Wilcoxon Rank Sum Test. - = not significant.

## Discussion

To our knowledge, this is among the first GWAS of an infectious disease and the first GWAS of KD. We have identified a number of novel variants using a staged study design and subsequent fine-mapping that are associated with KD susceptibility. These include variants within or close to genes that are functionally inter-related and that are plausible biological candidates in the KD pathogenesis. The magnitude of the effect sizes for KD susceptibility is comparable to that reported from other GWAS [17]. Fine-mapping of associated and replicated SNPs has focused on more frequent variants that lie in known genes. In eight of these sixteen genes, fine-mapping confirmed the association and identified one or more associated haplotype(s), which will form the basis of resequencing to identify the disease-modifying variants.

The assertion that these variants are in (or close to) biologically relevant loci involved in KD susceptibility is supported by; (i) identification of eight loci containing one or more independently associated haplotypes identified by GWAS, replicated in an independent family-based study and subsequently fine-mapped, (ii) the significant differential gene mRNA transcript abundance of 5 of the 7 blood-expressed fine-mapped genes during acute versus convalescent KD, and (iii) the gene network analyses that suggest biologically plausible functional relationships, which are extremely unlikely to have occurred by chance, exist between five of the associated loci.

We focused on fine-mapping of associated SNPs that lie either in or within 5 kb of known genes and had a MAF of >0.05 in HapMap. These data represent the most robust associations and we will therefore focus our discussion on those genes, where putative functional relationships were suggested by IPA. We used IPA in an unsupervised manner, allowing identification of gene-gene relationships without *a priori* assumptions. This analysis linked five of the eight genetically associated genes, of which four form a functionally closely related network linked to eight other nodes in a highly significant network. The gene network suggests possible mechanisms by which one or more infectious triggers may lead to dysregulated inflammation and apoptosis, and cardiovascular pathology.

Central to the putative gene network is *CAMK2D* (calcium/calmodulin-dependent protein kinase (CaM Kinase) II delta), whose expression was significantly down regulated during acute KD. *CAMK2D* encodes the δ-isoform of CaM kinase II (NP_001212), a ubiquitously expressed calcium sensitive serine/threonine kinase. The δ-isoform of CaM kinase II is the predominant form expressed in cardiomyocytes and vascular endothelial cells [18] and is involved in a number of pathophysiological processes that make it an attractive candidate in KD. In vascular endothelial cells CaM kinase II mediates nitric oxide (NO) production by endothelial synthase (NOS3, NP_000594) in response to changes in intracellular calcium and NO causes local vasodilatation [18]. In acute KD NO production is increased and NO metabolites decrease following successful treatment [19]. Following KD, especially where there has been overt CA damage, there is endothelial dysfunction and impaired vasodilatation, which can be restored after administration of antioxidants that may increase local availability of NO [20]. More chronically, NOS3 may become dysregulated (‘uncoupled’) and produce potentially harmful superoxide anions, resulting in chronic oxidant stress that is implicated in the pathogenesis of atherosclerosis [21]. In those with severe KD, NOS3 is expressed in coronary artery aneurysm tissue removed at surgery, and the tissue shows a pattern of senescence that is also typical of atherosclerosis [22].

Involvement of leukocyte expressed CaM kinase II in blood vessel damage and aneurysm formation, key features of KD, is also plausible. In human monocytes, CaM kinase II modulates tumor necrosis factor-induced expression of CD44 (NP_000601), which has a central role in leukocyte migration and extravasation at inflammatory sites [23]. CaM kinase II is also involved in disruption of the endothelial barrier following stimulation with agonists such as thrombin [24], whose levels may be increased following KD [25]. Disruption of barrier integrity in coronary arteries may contribute to leukocyte infiltration into the vessel wall, proteolysis of extracellular matrix proteins and the internal elastic lamina and subsequent coronary artery aneurysm formation, that is pathognomonic of KD [1].

In addition, CaM Kinase II regulates endotoxin- and TNF-mediated apoptosis in human promonocytic cells by regulating the anti-apoptotic gene *BIRC3* (Gene ID:330)[26]. Delayed apoptosis of leukocytes is characteristic of acute KD and may contribute to pathogenesis [27]. Intravenous immunoglobulin (IVIg), standard therapy for KD, induces apoptosis of neutrophils in acute KD [28]. In a genome-wide transcriptional study of KD, there was a marked over-representation of apoptosis regulatory genes [16].

Both CaM Kinase II and the product encoded by another fine-mapped gene *LNX1*, i.e. ligand of numb-protein X1 (NP_116011), interact with the NUMB family of proteins [29],[30]. Interestingly, one of the NUMB family members, *NUMBL* (numb homolog (Drosophila)-like, Gene ID:9253) lies in the same small haplotype block that has recently been associated with KD susceptibility by a linkage study and subsequent fine-mapping [12],[13]. The *NUMB* gene (Gene ID:8650) showed significantly higher transcript abundance during acute KD.

Both *LNX1* and *LNX2* (Gene ID:222484) (a closely related gene identified by the IPA network) encode proteins that bind the coxsackievirus and adenovirus receptor (*CXADR*, Gene ID:1525) [31]. *CXADR* is the receptor for coxsackievirus B3, which causes myocarditis in humans. The myocarditis can be prevented in animal models by antagonizing viral binding to CXADR (NP_001329.1) [32]. Coxsackievirus B3 has also been implicated in acute myocardial infarction [33]. Interestingly, the human endogenous retrovirus K protein Np9 also interacts with LNX1 [34] and therefore a number of viruses may theoretically bind the NUMB/CAR/LNX1 complex, leading to internalization and regulation of CAMK2D activity. This suggests a possible mechanism whereby more than one infectious trigger may result in cardiovascular damage in genetically susceptible individuals suffering from KD.

Other fine-mapped IPA-networked genes include *ZFHX3* (also known as *ATBF1*), which encodes a large enhancer-binding transcription factor that is known to be polymorphic [35] and interacts with a number of proteins, including PIAS3 (protein inhibitor of activated STAT, NP_006090) that inhibits STAT3 (signal transducer and activator of transcription-3, NP_644805) [36]. STAT3 is activated by interleukin 6 (IL6, NP_000591) a pro-inflammatory cytokine that is involved in early innate immune reactivity, as indicated by the high fever, acute phase response with increased levels of CRP (NP_000558), complement factors and fibrinogen, in the blood as well as the myriad of cellular markers altered in acute KD [37]. *ZFHX3* also interacts with MYH7 (myosin, heavy chain 7, cardiac muscle, beta, NP_000248), in which mutations are known to cause an inherited form of cardiomyopathy [38]. *CSMD1* (CUB and Sushi multiple domains 1), which is functionally related to CaM kinase II via histone deacetylase 4 (*HDAC4*, Gene ID:9759), may be associated with dampening the early phase of KD. *CSMD1* is located on chromosome 8, in a region that is hypervariable in humans and which contains numerous immune-related genes [39]. Activation of the classical complement pathway occurs in acute KD [40], and CSMD1 (NP_150094) is a complement regulatory protein that blocks the classical but not alternate complement pathway [41].

The functions of other fine-mapped genes are generally poorly understood. The most significantly associated gene (*NAALADL2*, N-acetylated alpha-linked acidic dipeptidase-like 2), which also showed the greatest change in transcript levels between acute and convalescent KD, is a large gene of 32 exons spanning 1.37 Mb. *NAALADL2* undergoes extensive alternative splicing leading to multiple 5′ and 3′ untranslated regions and variable coding sequences. Its function is largely unknown, but mutations in the gene may contribute to Cornelia de Lange syndrome (OMIM: 122470) [42].

Overall we have identified five genetically associated genes that also had significantly reduced transcript levels during acute KD, including three that are closely functionally related (Figure 4), suggesting that these genes may act together. This novel network may be distinct for KD as differences in transcript abundance in these genes have not been previously described as being part of a typical inflammatory response expressed in blood cells. Pathogen-specific host responses, identified by relative transcript abundance in the blood have been described for other infectious diseases [43].

Investigation of transcriptome abundance in whole blood rather than specific cell populations allows assessment of the entire peripheral blood transcriptome [43] and may be particularly informative in diseases such as KD, where an infectious trigger is implicated but remains unidentified [1]. In a genome-wide gene expression study of KD, variation in neutrophil and lymphocyte numbers, characteristic of acute KD [44],[45], were thought to account for approximately half of the variation in transcript abundance during the course of the KD illness [16]. Although we did not investigate individual cell populations in the current study, the data suggest that relative changes in transcripts reflect qualitative as well as quantitative differences. Given the enrichment of the expression profile with immune-related genes (selected on the basis of associated loci), the changes in mRNA may not be more numerous than those expected by chance and do not provide definitive proof for the gene-specific associations. While the number of subjects in our expression study is large enough to identify overall trends in the host response during KD, we are unable to comment on expression-related allelic association, which will be investigated in future studies. There is a suggestion from peripheral blood expression data in KD that ‘person-specific’ gene expression patterns, possibly reflective of underlying genetic variation, may be present [16]. Further investigation of the relationship between genomic associations and gene expression will be undertaken, although clearly genetic variants may be significantly associated with disease without resulting in alterations in gene expression.

Our sample of 893 cases represents a large genetic KD cohort drawn from a single ethnic group. KD shares many features of other infectious diseases of young children, including fever, rash and changes to the mucous membranes. There is no diagnostic test and laboratory parameters individually have insufficient sensitivity or specificity for diagnosis [1]. In all study cohorts we employed a conservative and widely accepted KD case definition in an attempt to maximize phenotypic homogeneity and diagnostic specificity. The similar ethnicity and ascertainment of KD cases in all cohorts reduces the risk of spurious associations [15].

Our methodological approach is consistent with current best practice recommendations in GWAS design and analysis, which are aimed to identify robust associations and reduce type 1 errors [15]: (i) the discovery and replication cohorts were recruited using very similar ascertainment techniques and drawn from predominantly Caucasian populations, with careful analysis to exclude cryptic population admixture in the discovery phase, which used a case-control design; (ii) the variants selected for replication were predominantly selected using single-point analysis, although we employed other models, including haplotypic analysis to maximize the informativeness of the initial GWAS data; (iii) we employed different genotyping technologies in each of the discovery, replication and fine-mapping stages to reduce spurious associations arising from genotyping errors; (iv) we limited our replication genotyping solely to variants identified in the discovery phase, as additional fine-mapping around associated variants in the replication phase may increase spurious associations [46]; (v) we used a staged study design to avoid conservative correction for multiple statistical comparisons that might mask associations of moderate effect size in this modestly sized sample; (vi) we present joint analysis of the discovery and replication data, rather than considering the replication data in isolation and; (vii) we have fine-mapped variants with a MAF>0.05 which lie within or close to known genes.

We are aware that the genomic coverage and power of the discovery phase of the GWAS were limited and calculate that the initial GWAS had only approximately 50% power to detect an OR of 2.0 with alpha<0.05. Our relatively modest sample size reflects the difficulties in recruiting for a relatively rare disease in which the phenotype is defined clinically. Our approach therefore aimed to reduce the risk of type I errors by ensuring that a large and independent replication cohort was included as part of the initial design, as we did not expect the associated variants to reach genome-wide significance, given the cohort size in the GWAS discovery phase [47]. It was therefore expected that neither previously reported and credible candidate gene associations in KD, such as *IL4* (Gene ID:3565) [48], *VEGFA* (Gene ID:7422) [49],[50], *CCR5* (Gene ID:1234) [51], and *MBL2* (Gene ID:4153) [52], nor the recently reported *ITPKC* variant [12] were replicated by the GWAS. Our study has failed to identify these and almost certainly other as yet unidentified variants that represent additional major determinants of KD susceptibility.

We have identified a number of novel associated SNPs, confirmed by fine-mapping, which lie within or close to previously unrecognized candidates for KD. The effect sizes, independent verification in different populations, differential transcript abundance and network analyses all indicate that at least a proportion of these variants represent novel genetic risk factors for KD. Some of the associated genes may interact to mediate the deleterious effects of infection-driven inflammation on the cardiovascular system. Further characterization of the associated genes and their functional interactions may lead to the identification of novel diagnostic and therapeutic targets in KD and may be informative about early pathogenic processes in other cardiovascular diseases.

## Materials and Methods

### Study Design

We used a staged study design with an initial GWAS, an exact replication phase in an independent cohort and subsequent fine-mapping of common variants lying within or near known genes. We performed the initial GWAS analysis in a Dutch Caucasian case-control sample (the ‘discovery phase’) and re-tested the most significantly associated SNPs and haplotypes in an independent sample of KD trios from Australia, the US and the UK, using a different genotyping technology (the ‘replication phase’). Finally, a fine-mapping stage including sixteen replicated genes was performed in a subset of samples from discovery and replication phases, again using a different genotyping platform.

### Phenotypic Definition and Case Ascertainment

KD was defined by the presence of prolonged fever, together with at least four of the five classical diagnostic criteria [53]. Children with at least five days of fever and two diagnostic criteria with echocardiographic changes of coronary artery damage during the acute and/or convalescent phases of KD were also included, as these coronary artery manifestations are pathognomonic for KD [53]. Details of clinical symptoms of our study group can be found on Table S5. Cases of incomplete KD, who have fever, less than 4 diagnostic criteria and no coronary artery manifestations (who constitute approximately 15% of KD cases receiving clinical treatment [54]), were excluded, to maximise the homogeneity of the clinical phenotype.

In all cohorts clinical and laboratory data were obtained directly from patient medical files and supplemented with parental questionnaires. Phenotypic data were reviewed in all cases by experienced pediatricians. Ethical approval was obtained from the appropriate national and regional institutional review boards for each study population (Academic Medical Center [AMC], Amsterdam (Dutch cohort), UK Multi-Centre Research Ethics Committee (UK cohort), each participating tertiary pediatric hospital's ethics committee (Australian cohort) and the University of California at San Diego (US cohort)). Informed consent and assent as appropriate were obtained from participating families.

### Subjects

The initial GWAS was performed on 119 Dutch Caucasian KD cases and 135 healthy controls. The cases were identified by collaborating pediatricians and sent for cardiological evaluation during the acute stage and subsequent follow-up to the AMC. The controls were unrelated adult Caucasian blood donors residing in the same geographical area. Ethnicity was determined by self or parental ethnic identification. Assessment for possible population stratification was performed with Eigenstrat [14]. Principal component analysis was applied to the genotype data to infer the axes of variation. We used the top two principal components to identify outliers. Any sample with principal component exceeding six standard deviations from the mean was identified as an outlier. This process was repeated five times. A lambda genomic control (λ_GC_), representation of stratification estimated after dividing the median (chi-square) by 0.456, was calculated before and after running Eigenstrat. We observed a λ_GC_ = 1.18 before removal of potential outliers. After running Eigenstrat (sigma 6.0, 5 iterations, n = 10 individuals removed) λ_GC_ dropped to 1.1. Dividing chi-square values by λ_GC_ we were able to correct for possible existence of population admixture (Table 2). Sample duplication and family relationships were assessed by RelPair [55].

A second independent cohort, which consisted of 583 KD-affected families, including complete and incomplete trios, from Australia, the US and the UK, was used to replicate the most significantly associated variants identified in the GWAS. Family KD cases in each country were identified from pediatric hospital databases, through KD parent support groups and through media releases. Biological parents (where available) and unaffected siblings (to reconstruct missing parental genotypes) were recruited.

A subset of samples from our GWA and follow up cohorts was genotyped in a fine-mapping experiment. Due to limitations in DNA template we could include ∼85% of the original samples. However, a new set of 493 samples were added in the case-control (N = 247) and family-based cohorts (N = 246). A principal component analysis comparing Hapmap populations with our cohort was applied to the genotype data of the case-control cohort to infer the axes of variation (Figure S1). After removal of potential outliers (N = 113) λ_GC_ was 1.06. Allele chi-square values were divided by λ_GC_ and corrected p-values are reported on Table 4.

### DNA and RNA Collection and Extraction

Blood was collected into EDTA (Dutch, UK and US cohorts) and ADC (Australian cohorts, on whom Buffy coats were separated). Shed buccal cells were collected as previously described [51]. Genomic DNA was extracted by standard protocols. DNA quality was assessed by visual inspection after running 1.2% agarose gels and by calculating absorbance ratio OD_260 nm/280 nm_. DNA quantification was measured using Picogreen dsDNA reagent. Degraded samples or those with low DNA concentration were excluded.

RNA samples from acute KD cases were obtained prior to intravenous immunoglobulin treatment and again during convalescence (within one year n = 17, after one year n = 10) from 27 US children who fulfilled the KD case definition. These KD patients were also analyzed as part of the replication cohort. Whole blood was collected into the PaxGene tubes, according to manufacturer's instructions and RNA was extracted and stored at −80°C for batch analysis. RNA quantification was performed by optical density (260 nM).

### Genotyping in GWAS and Follow-Up Studies

We genotyped the Dutch case-control samples using the Affymetrix 250 K NSP chip in accordance with the manufacturer's instructions. Samples with call rates below 93% at p = 0.33 after running a BRLMM algorithm (Bayesian Robust Linear Model with Mahalanobis distance classifier, Affymetrix), were re-hybridized and were excluded from further analysis if they failed to achieve the established threshold.

Associated individual SNPs from the GWAS were ranked by nominal significance using a combination of allelic association, Armitage trend test and a recessive-dominant model. SNPs that deviated significantly from HWE in the control group after applying a Bonferroni correction, or failed genotyping QC (call rate<93%, or monomorphic) were excluded from further analysis. The 1176 most significantly associated SNPs were selected for genotyping in the Australian, UK and US trios by a custom Illumina Oligo Pool Assay. For 28 SNPs genotyping assays could not be designed, leaving 1148 SNPs for the follow-up study. Families were excluded if no familial relationship was detected by RelPair [55], if there was sample duplication, if the proband genotype was unavailable or if they had more than 3 Mendelian errors. Analysis of the successfully genotyped SNPs was performed with Illumina BeadStudio software. In addition, haplotypic analysis of the GWA data was performed, using a multi-marker sliding window [56]. Genotyping of all 166 SNPs identified by haplotypic analyses was performed by Sequenom iPLEX in the family-based follow-up cohort.

### Identifying Associated Genes

For each disease-associated polymorphism verified in the follow-up family study, we investigated whether the variant was within an annotated gene. When the SNP was in an inter-genic region, we analyzed the closest annotated genes situated within 50 kb up- and downstream.

### Fine-Mapping of Associated Variants

Replicated polymorphisms with allele frequencies over 5%, located within genes or five kb up or downstream of a gene were selected for fine-mapping. Sixteen genes fulfilled criteria (Table S3). Using a SNP-tagging approach (r^2^≥0.8) we selected 1052 SNPs from Hapmap for genotyping with Illumina ISelect Infinium. After standard quality control (call rate<93%, minor allele frequency below 0.01) 1,003 SNPs were included in the association analysis (Table S3).

### Gene Ontology Clustering

Associated genes were investigated for gene ontology information by *Ingenuity Pathways Analysis* (IPA) software (Ingenuity Systems, www.ingenuity.com) using an unsupervised analysis. To start building networks, IPA queries the Ingenuity Pathways Knowledge Base for interactions between identified ‘Focus Genes’, in our case genes detected in the GWAS, and all other gene objects stored in the knowledge base, to generate a set of networks with a maximum network size of 35 genes/proteins. Networks are displayed graphically as genes/gene products (‘nodes’) and the biological relationships between the nodes (‘edges’). All edges are supported by at least one reference from the literature, or from canonical information stored in the Ingenuity Pathways Knowledge Base. In addition, IPA computes a score for each network according to the fit of the user's set of significant genes. The score, representing the –log (p-value), indicates the likelihood of the Focus Genes in a network from Ingenuity's knowledge base being found together due to random chance.

### Gene Expression

Genes corresponding to associations or identified by IPA were investigated for differential expression (between acute and convalescent KD) using Taqman low density array (TLDA; Applied Biosystems, Foster City, CA, USA) on 2 µg total RNA as per manufacturer's instructions. Briefly, 2 µg total RNA was reverse transcribed using High-Capacity cDNA Archive Kit (Applied Biosystems, Foster City, CA, USA). Reverse-transcriptase reaction was performed at 25°C for 10 min and then 37°C for 2 hours followed by 85°C for 5 seconds. cDNA converted from 0.1 ug RNA was resuspended in 50 µl buffer and was added to 50 µl TaqMan Universal Master Mix (2×) (Applied Biosystems) then immediately loaded into a Micro Fluidic Card (3M Company, Applied Biosystems). The card was spun twice at 1200 rpm for 1 min each time to distribute the PCR mix into the wells of the card before sealing and loading into the ABI 7900HT sequence detection system. Default thermal cycling conditions were used (50°C for 2 min with 100% ramping, 94.5°C for 10 min with 100% ramping, and finally 40 cycles of 97°C for 30 sec with 50% ramping and 59.7°C for 1 min with 100% ramping) and data were normalized for RNA loading levels by using 18 s quantitation as a reference and exported using SDS RQ Manager software (Applied Biosystems, Foster City, CA, USA). Relative (RQ) levels were exported and analyzed for significance using the Wilcoxon Rank Sum Test (assuming the data are non-normally distributed). Fold-change analysis was based on median levels for the acute stage samples over the convalescence samples.

### Statistical Analyses

In the initial GWAS, HWE in the control group and allelic association analysis were calculated using *HelixTree v4.4.1* (GoldenHelix Inc., Bozeman, MT, United States) and *Exemplar* (Sapio Sciences, LLC, York, PA, United States). Allelic P-values were calculated by means of a 2×2 chi-square table and an Armitage trend test was used to derive genotypic p-values. Genotype association analysis and odds ratios were calculated using *Exemplar*. A two-sided Fisher's exact test was used when counts in any cell fell below five. Allelic analysis of the X chromosome SNPs was performed with *Haploview v3.31* [57] and a likelihood ratio test was applied to calculate genotypic associations. Quantile-quantile (Q-Q) plots were constructed by ranking a set of values of –log p-value and plotting them against their expected values. Deviations from the line of equality indicated either that the theoretical distribution was incorrect, or that the sample was contaminated with values generated in some other manner (for example, by a true association). The family-based association analysis was performed using FBAT [58], allowing transmission disequilibrium analysis in extended families. Combined p-values and odds ratios were calculated by Fisher's combined probability test which allows pooled information across several tests that share the same null hypothesis [59].

### Haplotypic Analysis

Analysis of haplotypic associations was performed using a recently described method, the VSSWRR (Variable-Sized Sliding Window via Regularized Regression), a haplotype analysis method for population-based case-control association studies [56]. It uses a variable-size sliding window where the maximum window size is determined by local haplotype diversity and the sample size and a combined analysis of all the haplotypes of different lengths (up to the maximum window size) at the same starting position is performed using L_1_-regularized regression method, adjusting for the dependency and complementariness among the haplotypes. It allows efficient management of a large number of haplotypes and is powerful in the detection of disease associations [56].

We used the Haplotype Relative Risk (HRR) method for testing for LD between marker genotypes and disease phenotypes for the trios (affected offspring and parents) in the fine-mapping study design [60]. For testing LD this method compares the transmitted parental alleles to affected offspring to those which are not transmitted. In many of the genetic association studies complete genotypes for both the parent may not be available. Hence to use partial information available in the affected child in trios in which both single and two parent genotypes are missing, Guo et al (2005) extended the HRR methods using Expectation Maximization (EM) algorithm. Assuming that the parental genotypes are missing at random they use EM algorithm to estimate the proportion of parents who transmitted a specific allele and non-transmitted the other allele. If there is not a severe admixture among the families, through simulations Guo et al. (2005) have demonstrated that the EM-HRR gained power by including the families with the affected child for which single or both the parental genotypes are missing [61].

## Supporting Information

Figure S1 Principal Component Analysis of Fine-mapping Case-Control Group (372 case-control) and Hapmap populations (206 individuals). A principal component analysis comparing Hapmap populations with our cohort was applied to the genotype data of the case-control cohort to infer the axes of variation showing our study group (light blue and light orange dots) clustering with CEU population (yellow dots) and clearly separated from Asians (dark blue and white dots) and Africans (green dots).
(1.94 MB TIF)

Figure S2 Linkage Disequilibrium Plot of a Region Containing the Most Significant P-values of *ZFHX3*. The upper portion shows -log (p-values). SNPs in high LD (r^2^>0.8) with polymorphisms in our initial GWA are highlighted with blue boxes and blue dots. D′ values indicate inside each diamond. Red diamonds without a number represent D′ = 1.
(4.16 MB TIF)

Figure S3 Linkage Disequilibrium Plot of a Region Containing the Most Significant P-values of *DGKB*. The upper portion shows -log (p-values). SNP replicated from our initial GWA (rs196751) is highlighted with a blue box and blue dot. D′ values indicate inside each diamond. Red diamonds without a number represent D′ = 1.
(4.01 MB TIF)

Figure S4 Linkage Disequilibrium Plot of *CAMK2D*. The upper portion shows -log (p-values). D′ values indicate inside each diamond. Red diamonds without a number represent D′ = 1.
(0.73 MB TIF)

Figure S5 Linkage Disequilibrium Plot of *CSMD1*. The upper portion shows -log (p-values). D′ values indicate inside each diamond. Red diamonds without a number represent D′ = 1.
(0.08 MB TIF)

Figure S6 Linkage Disequilibrium Plot of *LNX1*. The upper portion shows -log (p-values). D′ values indicate inside each diamond. Red diamonds without a number represent D′ = 1.
(0.21 MB TIF)

Figure S7 Linkage Disequilibrium Plot of *PPPR114C*. The upper portion shows -log (p-values). D′ values indicate inside each diamond. Red diamonds without a number represent D′ = 1.
(0.52 MB TIF)

Figure S8 Linkage Disequilibrium Plot of *TCP1*. The upper portion shows -log (p-values). D′ values indicate inside each diamond. Red diamonds without a number represent D′ = 1.
(0.04 MB TIF)

Table S1 Chromosomal Location and Allele Frequencies of 1,116 Genotyped SNPs in the Follow-Up Study.
(0.17 MB XLS)

Table S2 Chromosomal Location, Allele Frequencies and Haplotype Blocks of 137 Genotyped SNPs in the Follow-Up Study Derived from Haplotype Analysis.
(2.44 MB XLS)

Table S3 Chromosomal Location, Gene and Consequence of 1,003 SNPs Successfully Genotyped in Fine-Mapping Stage.
(0.12 MB XLS)

Table S4 Associated Allele, Minor Allele Frequencies in Selected Populations and Ancestral Alleles of 46 Associated SNPs from the Fine-mapping Stage.
(0.03 MB XLS)

Table S5 Detailed Description of Patients Fulfilling Classical Symptoms in Kawasaki Disease (individuals are included only if complete clinical data were available).
(0.02 MB XLS)
