# Supplementary figures and images for: A Genome-Wide Association Study Identifies Novel and Functionally Related Susceptibility Loci for Kawasaki Disease
Source: PLoS Genet. 2009 Jan 9;5(1):e1000319. doi: 10.1371/journal.pgen.1000319 (PMC2607021; doi:10.1371/journal.pgen.1000319)

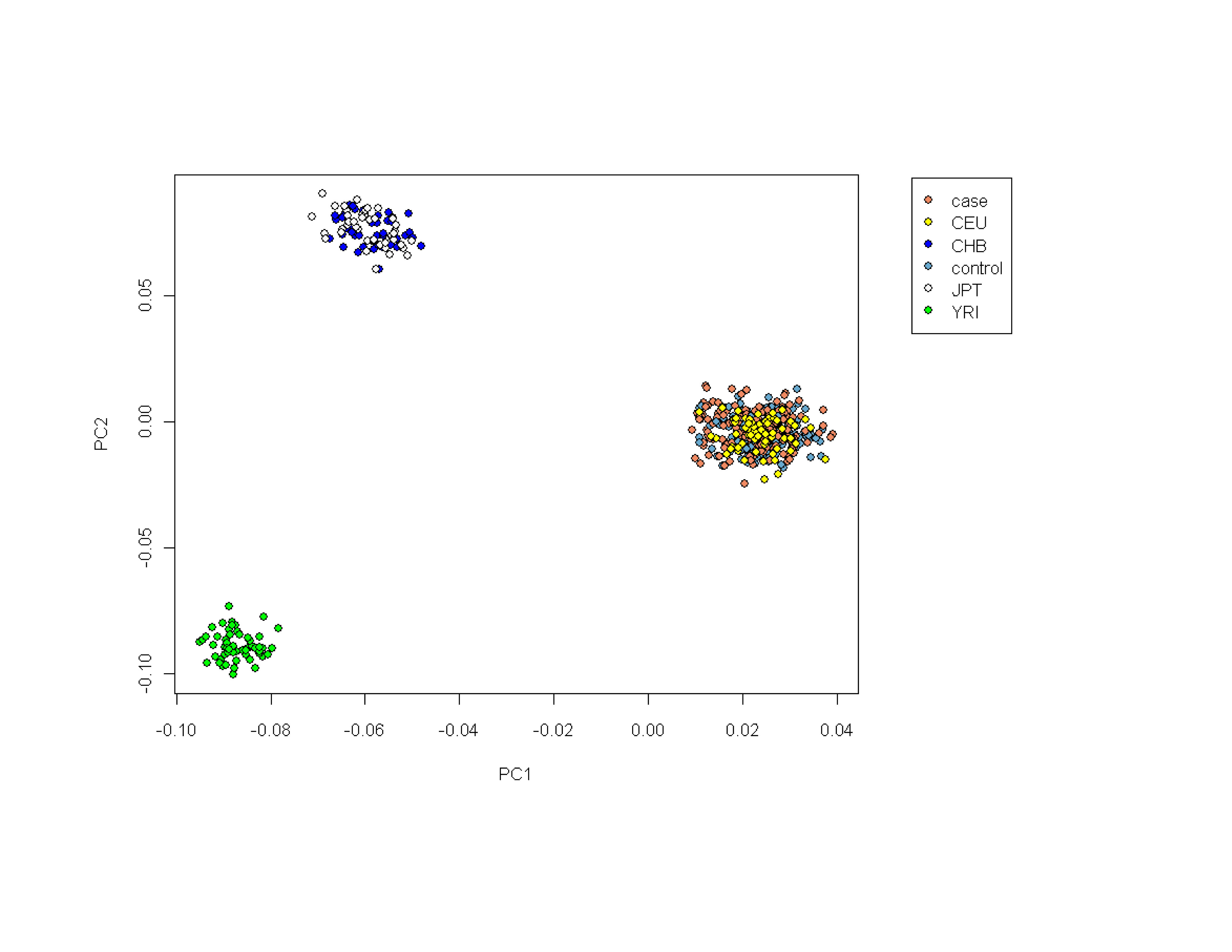

Supplement: Figure S1 — Principal Component Analysis of Fine-mapping Case-Control Group (372 case-control) and Hapmap populations (206 individuals). A principal component analysis comparing Hapmap populations with our cohort was applied to the genotype data of the case-control cohort to infer the axes of variation showing our study group (light blue and light orange dots) clustering with CEU population (yellow dots) and clearly separated from Asians (dark blue and white dots) and Africans (green dots). (1.94 MB TIF) [file pgen.1000319.s001.tif]

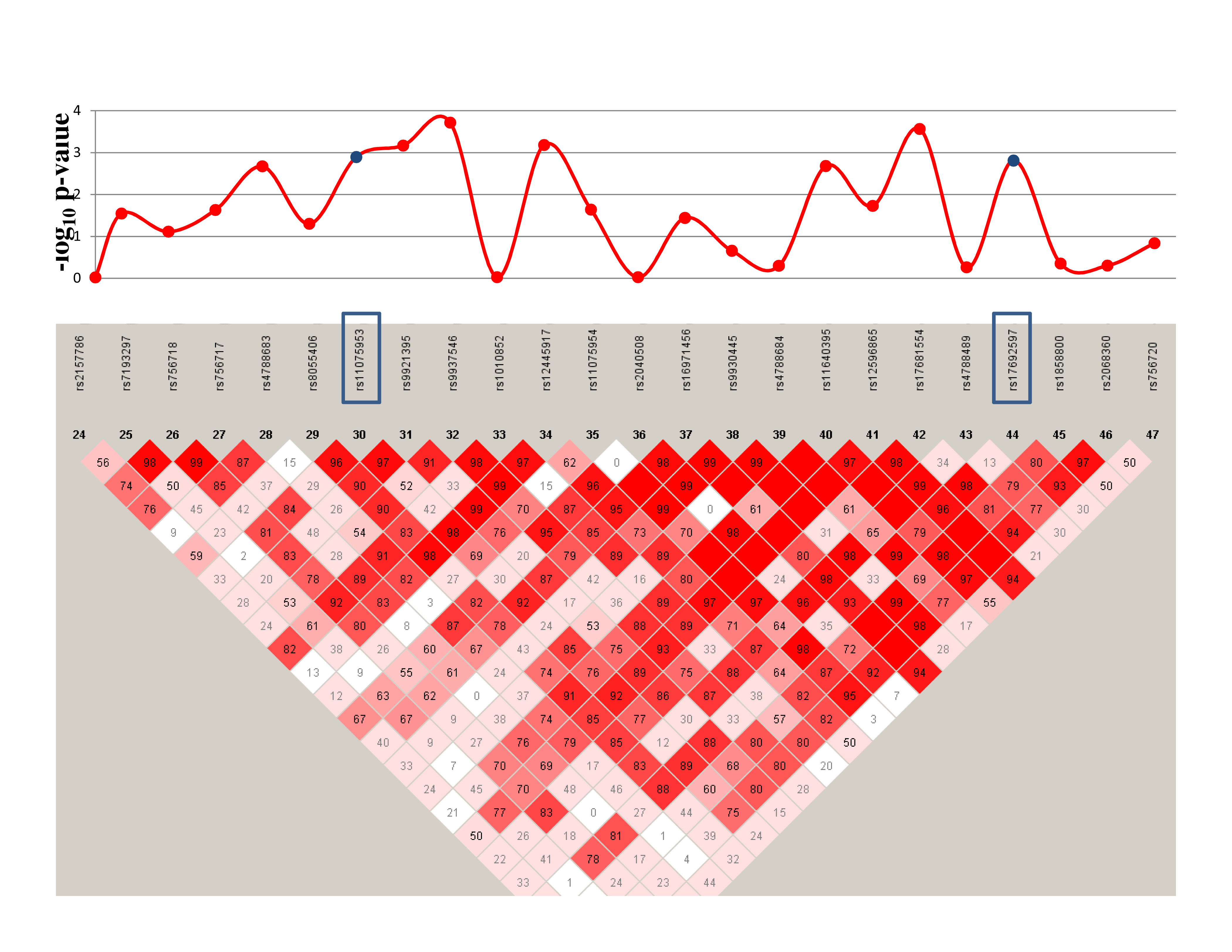

Supplement: Figure S2 — Linkage Disequilibrium Plot of a Region Containing the Most Significant P-values of ZFHX3. The upper portion shows -log (p-values). SNPs in high LD (r2>0.8) with polymorphisms in our initial GWA are highlighted with blue boxes and blue dots. D′ values indicate inside each diamond. Red diamonds without a number represent D′ = 1. (4.16 MB TIF) [file pgen.1000319.s002.tif]

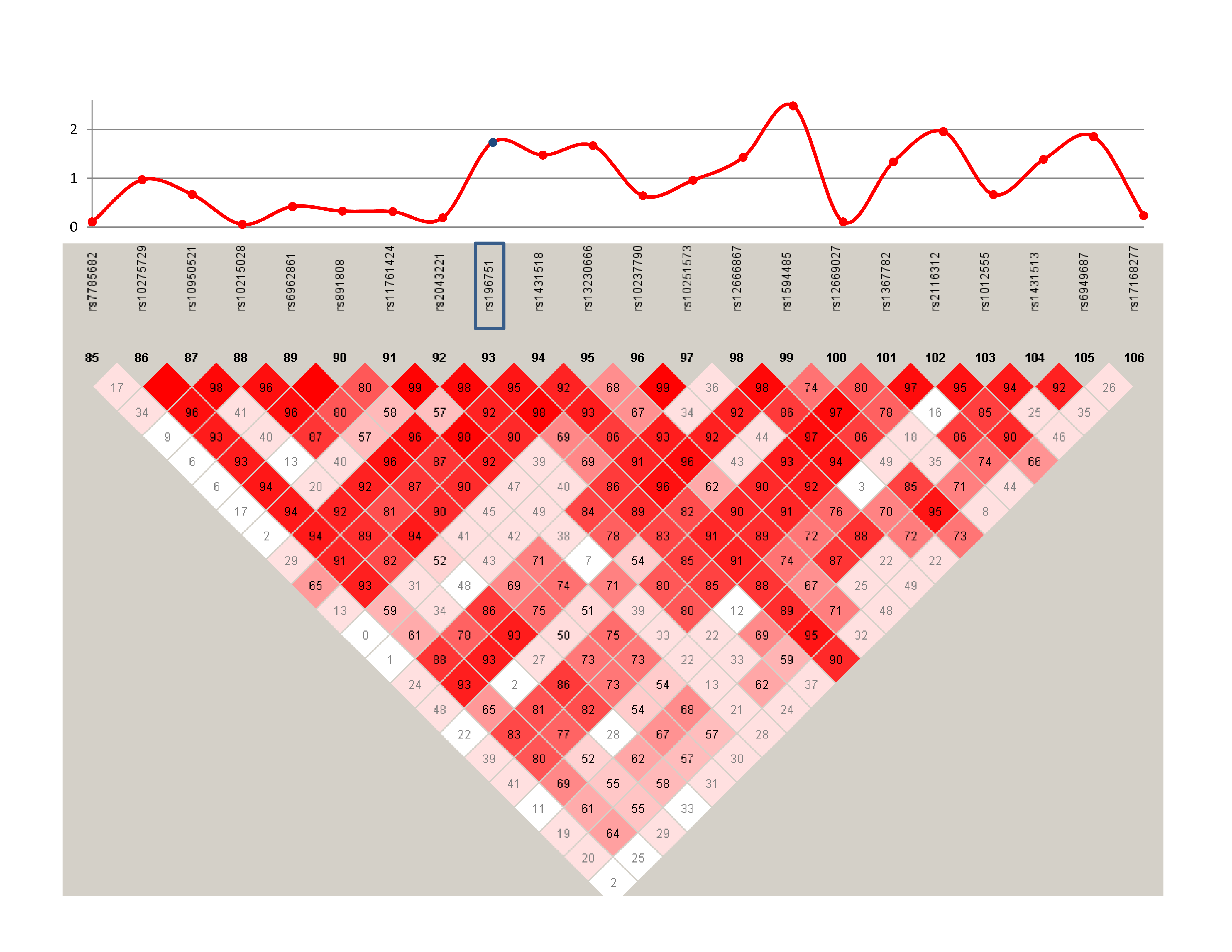

Supplement: Figure S3 — Linkage Disequilibrium Plot of a Region Containing the Most Significant P-values of DGKB. The upper portion shows -log (p-values). SNP replicated from our initial GWA (rs196751) is highlighted with a blue box and blue dot. D′ values indicate inside each diamond. Red diamonds without a number represent D′ = 1. (4.01 MB TIF) [file pgen.1000319.s003.tif]

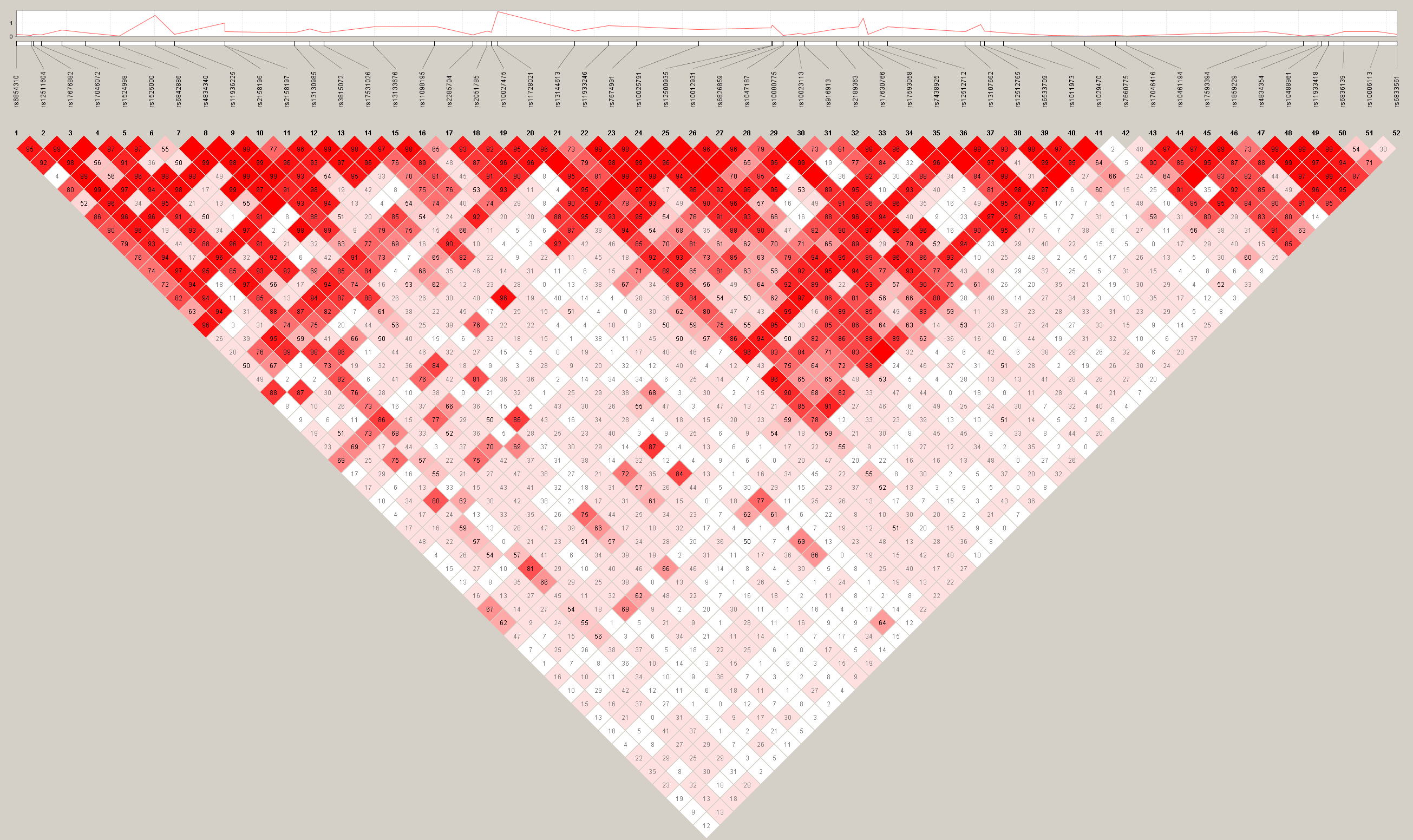

Supplement: Figure S4 — Linkage Disequilibrium Plot of CAMK2D. The upper portion shows -log (p-values). D′ values indicate inside each diamond. Red diamonds without a number represent D′ = 1. (0.73 MB TIF) [file pgen.1000319.s004.tif]

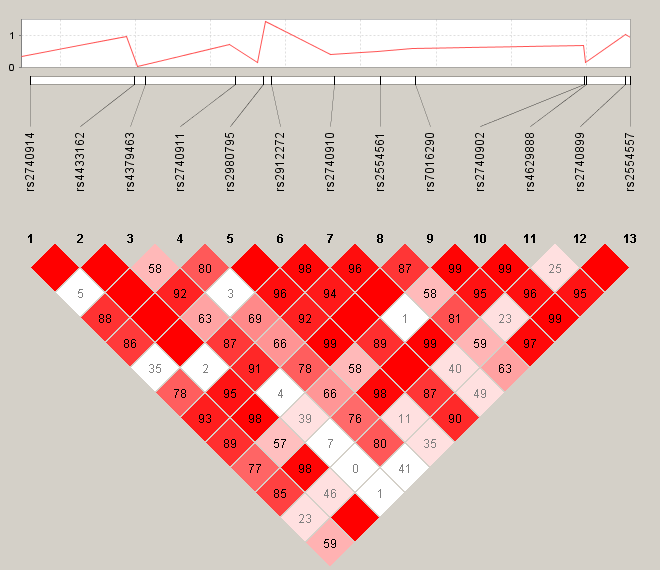

Supplement: Figure S5 — Linkage Disequilibrium Plot of CSMD1. The upper portion shows -log (p-values). D′ values indicate inside each diamond. Red diamonds without a number represent D′ = 1. (0.08 MB TIF) [file pgen.1000319.s005.tif]

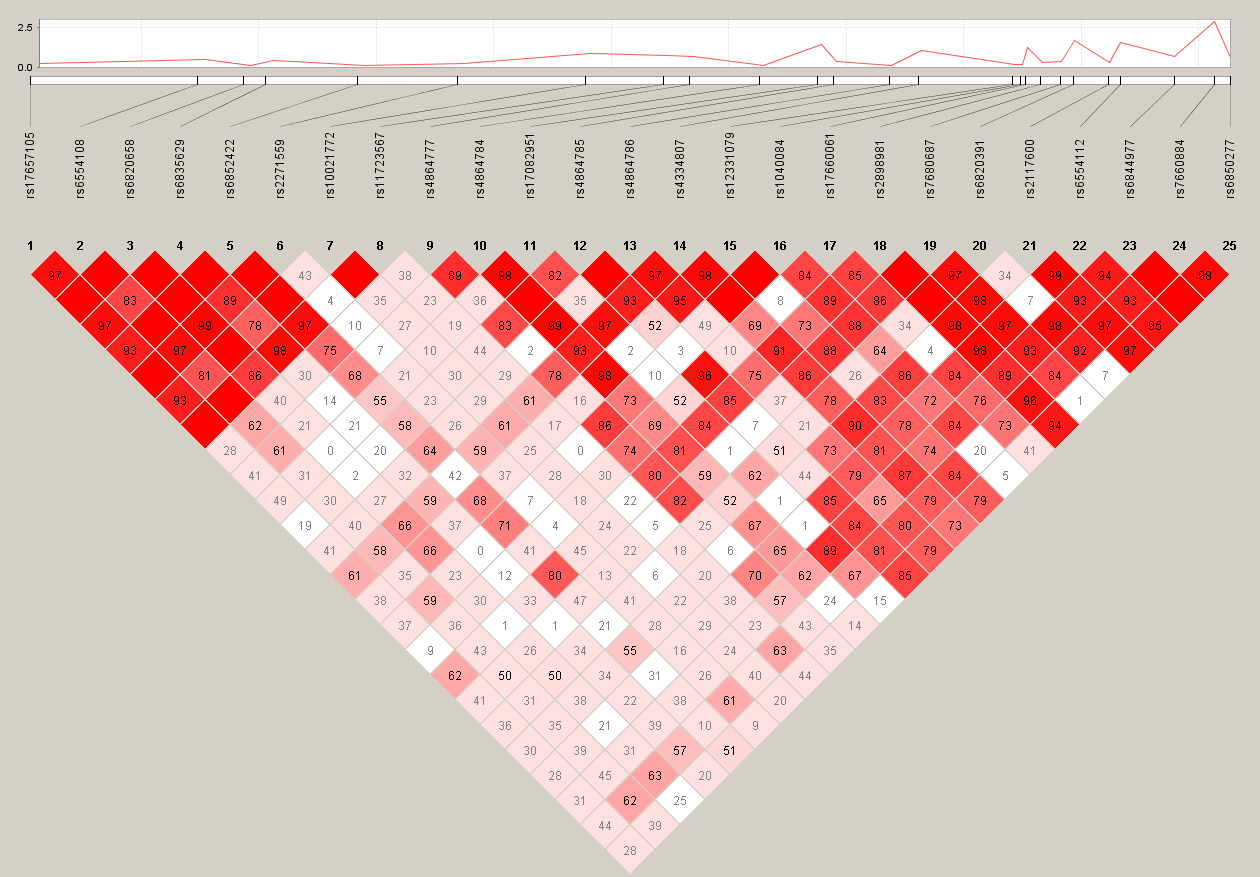

Supplement: Figure S6 — Linkage Disequilibrium Plot of LNX1. The upper portion shows -log (p-values). D′ values indicate inside each diamond. Red diamonds without a number represent D′ = 1. (0.21 MB TIF) [file pgen.1000319.s006.tif]

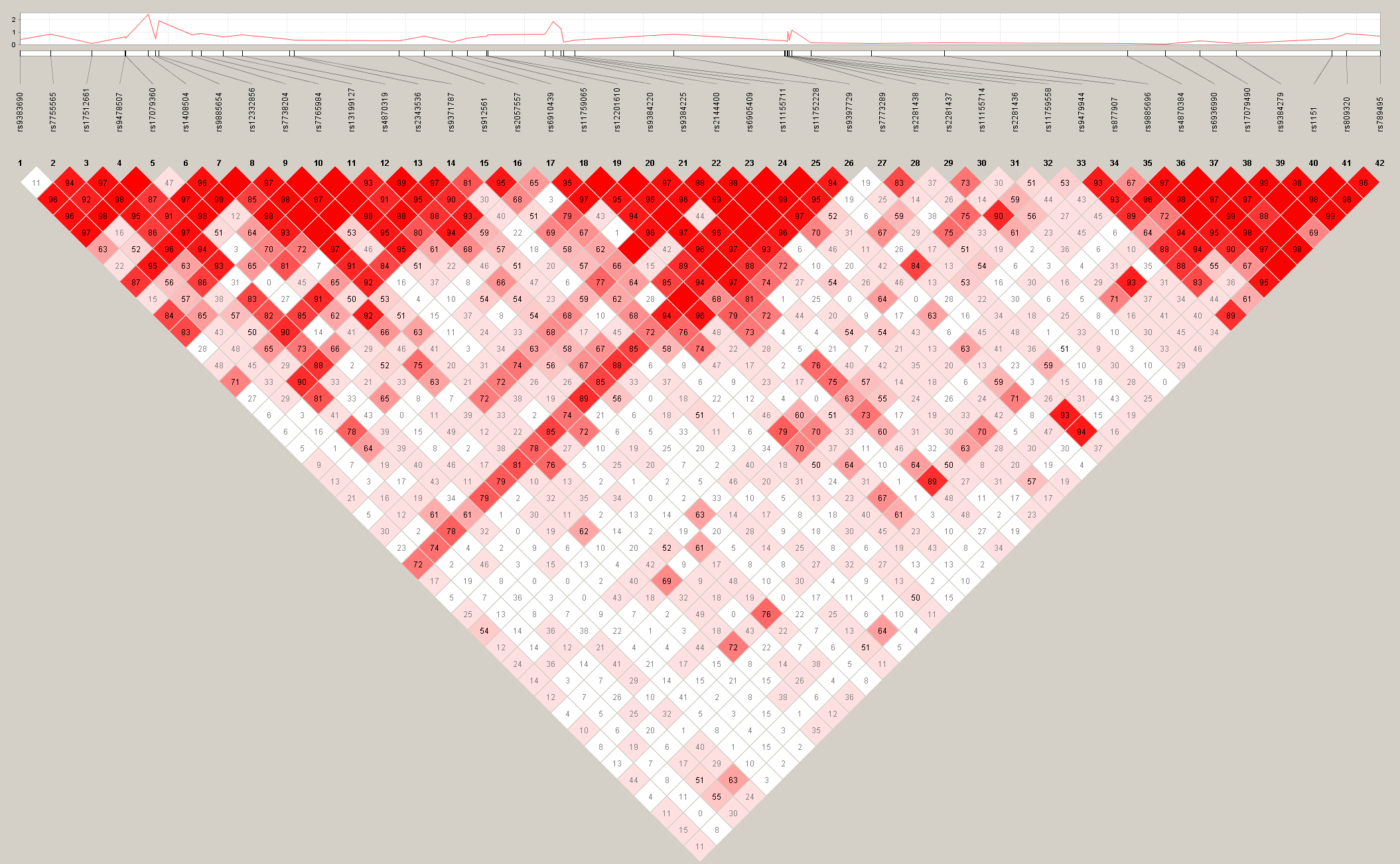

Supplement: Figure S7 — Linkage Disequilibrium Plot of PPPR114C. The upper portion shows -log (p-values). D′ values indicate inside each diamond. Red diamonds without a number represent D′ = 1. (0.52 MB TIF) [file pgen.1000319.s007.tif]

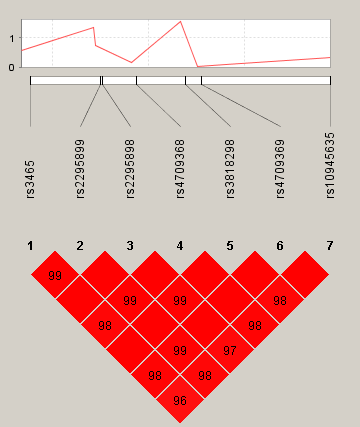

Supplement: Figure S8 — Linkage Disequilibrium Plot of TCP1. The upper portion shows -log (p-values). D′ values indicate inside each diamond. Red diamonds without a number represent D′ = 1. (0.04 MB TIF) [file pgen.1000319.s008.tif]
